# Linagliptin and cardiovascular outcomes in type 2 diabetes after acute coronary syndrome or acute ischemic stroke

**DOI:** 10.1186/s12933-017-0655-y

**Published:** 2018-01-04

**Authors:** Yan-Rong Li, Sung-Sheng Tsai, Dong-Yi Chen, Szu-Tah Chen, Jui-Hung Sun, Hung-Yu Chang, Miaw-Jene Liou, Tien-Hsing Chen

**Affiliations:** 1 Division of Endocrinology and Metabolism, Department of Internal Medicine, Chang Gung Memorial Hospital, Taoyuan, Taiwan; 2 Division of Cardiology, Department of Internal Medicine, Chang Gung Memorial Hospital, Taoyuan, Taiwan; 3 Division of Cardiology, Department of Internal Medicine, Chang Gung Memorial Hospital, No.222, Maijin Road, Keelung, Taiwan; 4 Chang Gung University College of Medicine, Taoyuan, Taiwan

**Keywords:** Linagliptin, Dipeptidyl peptidase-4 (DPP-4) inhibitor, Type 2 diabetes mellitus, Acute coronary syndrome, Acute ischemic stroke, Cardiovascular outcome

## Abstract

**Background:**

The cardiovascular safety and efficacy of linagliptin, a dipeptidyl peptidase-4 inhibitor, in patients with type 2 diabetes mellitus (T2DM) after acute coronary syndrome (ACS) or acute ischemic stroke (AIS) are unclear. The aim of our real-world cohort study was to evaluate the cardiovascular outcomes of linagliptin in patients with T2DM after ACS or AIS.

**Methods:**

An open observational noncrossover retrospective cohort study was conducted between June 1, 2012 and December 31, 2013 utilizing Taiwan National Health Insurance Research Database. A total of 1203 patients with T2DM after ACS or AIS were selected as the study cohort. Cardiovascular safety and efficacy of linagliptin were evaluated by comparing outcomes of 401 subjects receiving linagliptin after ACS or AIS to 802 matched control subjects not receiving any incretin-based therapy after ACS or AIS. The primary composite outcome included cardiovascular death, non-fatal myocardial infarction and non-fatal ischemic stroke.

**Results:**

The primary composite outcome after 15-month follow-up was 7% (28 patients) in the linagliptin group compared with 6.1% (49 patients) in the control group [hazard ratio (HR) 1.06; 95% confidence interval (CI) .66–1.68]. The linagliptin group also had similar risks of all-cause mortality, hospitalization for heart failure, percutaneous coronary intervention and coronary artery bypass grafting compared to the control group in terms of the secondary outcomes.

**Conclusions:**

In T2DM patients after ACS or AIS, treatment with linagliptin was not associated with increased risks of cardiovascular death, non-fatal myocardial infarction, or non-fatal ischemic stroke.

## Background

Type 2 diabetes mellitus (T2DM) is considered as an equivalent of coronary heart disease [1] with a twofold higher risk both for ischemic stroke [2] and mortality [3] compared to those without T2DM. More than 50% of deaths in diabetic patients were attributed to cardiovascular complications [4]. Although improvement of glycemic control reduces the risk of microvascular complications in patients with T2DM [5], several clinical trials have shown no benefit of reducing macrovascular risks with intensively glycemic control, especially for T2DM patients at high risk for cardiovascular events [6–9]. Another issue of concern raised by the Food and Drug Administration (FDA) in December 2008 is that the anti-diabetic agents should not be attributed to increase adverse events of cardiovascular diseases [10]; thus, specific requirements for cardiovascular safety assessment before and after the approval must be met [11].

Four kinds of dipeptidyl peptidase-4 (DPP-4) inhibitors (saxagliptin, alogliptin, sitagliptin, and linagliptin) are available for the treatment of T2DM in the United States. Vildagliptin is approved for use in many countries but not in the United States. Among them, three previous cardiovascular outcome trials of DPP-4 inhibitors (Saxagliptin Assessment of Vascular Outcomes Recorded in Patients with Diabetes Mellitus-Thrombolysis in Myocardial Infarction 53 (SAVOR-TIMI 53), Examination of Cardiovascular Outcomes With Alogliptin Versus Standard of Care (EXAMINE), Trial Evaluating Cardiovascular Outcomes with Sitagliptin (TECOS)) suggested no increased risks of cardiovascular death, myocardial infarction and stroke with a short-term use (a median follow-up of 1.5–3 years) of DPP-4 inhibitors [12–14], but an increased risk of heart failure with specific DPP-4 inhibitors were observed [12, 15]. The FDA on April 5, 2016 recommended the discontinuation of the use of saxagliptin or alogliptin in patients with T2DM if there is any evidence of emerging heart failure [16]. As a result, it seems to be not a class effect of DPP-4 inhibitors in the view of increasing heart failure [17].

Linagliptin exerts anti-hyperglycemic effects by increasing and prolonging active glucagon-like peptide-1 (GLP-1) levels which leads to increase pancreatic insulin secretion and suppressing pancreatic glucagon secretion [18]. The features of linagliptin are xanthine-based compounds which differ from the other DPP-4 inhibitors with higher selectivity for DPP-4 versus DPP-8 (40,000-fold) and DPP-9 (> 10,000-fold) [18, 19] and no dosage adjustment in renal insufficiency including end-stage renal disease because of primarily eliminated via the enterohepatic system [20]. Therefore, it is not clear whether the pharmacological differences between linagliptin and other DPP-4 inhibitors may result in differences in the cardiovascular safety profile. Two ongoing cardiovascular outcome trials for linagliptin (the Cardiovascular and Renal Microvascular Outcome Study with Linagliptin in Patients with Type 2 Diabetes Mellitus (CARMELINA) and the Cardiovascular Outcome Study of Linagliptin Versus Glimepiride in Patients with Type 2 Diabetes (CAROLINA)) estimate the completion of the studies by January 2018 [21] and March 2019 [22], respectively. However, one caveat to be considered in both clinical trials of CARMELINA and CAROLINA is that patients with recent acute coronary syndrome or acute stroke were excluded from these clinical studies. Therefore, there is still limited post-marketing data about safety and efficacy of linagliptin in patients at very high cardiovascular risks [23, 24]. Moreover, some evidence suggested that linagliptin may have neuroprotective effects and be associated with significantly fewer events of stroke [25, 26]. As a result, the aim of our real-world cohort study was to evaluate the cardiovascular outcomes of linagliptin T2DM patients after acute coronary syndrome (ACS) or acute ischemic stroke (AIS).

## Methods

### Data source

The National Health Insurance (NHI) program covers more than 99% of the 23 million people in Taiwan. All the submitted standardized information and data on healthcare service are prospectively recorded by the NHI Research Database (NHIRD). The diagnoses are registered using the International Classification of Diseases, Ninth Revision, Clinical Modification (ICD-9-CM) codes. The NHI Bureau routinely and comprehensively performs the validation of accurate records of beneficiaries, including ambulatory visits, inpatient care, disease diagnosis codes and medication prescriptions from the NHIRD data [27–30]. This kind of nationwide database from the NHIRD is important and contributory to many large population-based studies [31]. The personal informations and records of the patients were de-identified before analysis to ensure patients’ anonymity. This study was approved by the Ethics Institutional Review Board of Chang Gung Memorial Hospital (201701079B1).

### Patient enrollment and exclusion criteria

**Fig. 1:**
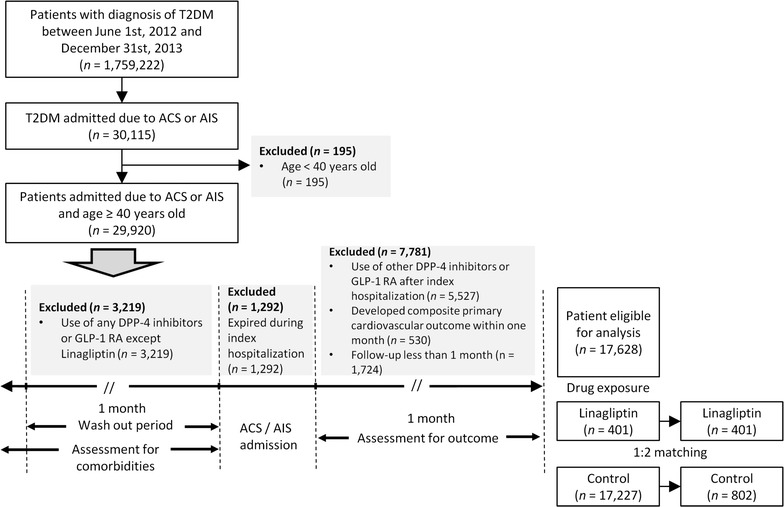
Flow chart of study subjects selection

This open observational noncrossover retrospective cohort study was derived from the NHIRD. Between June 1, 2012 and December 31, 2013, a total of 1,759,222 T2DM (ICD-9-CM code: 250) patients were initially enrolled and after applying exclusion criteria, a final total of 1203 T2DM patients who were hospitalized for ACS (ICD-9-CM codes: 410–411) or AIS (ICD-9-CM codes: 433–435) were included in our study (Fig. 1). The index hospitalization was defined as the date on which patient was admitted for ACS or AIS. In addition to identifying T2DM patients using ICD-9-CM codes, we defined T2DM patients with at least 90 days of prescribed oral hypoglycemic agents or insulin injection within 1 year of the index hospitalization.

The follow-up period was based upon the index hospitalization to date of death, loss of follow-up, or until December 31, 2013. Patients’ baseline characteristics, comorbidities, medication prescription and previous medical procedures, such as percutaneous coronary intervention (PCI), coronary artery bypass grafting (CABG) and carotid stenting were identified. Patients were excluded if they met any of the following criteria: (1) age below 40 years; (2) use of any DPP-4 inhibitors except for linagliptin or any glucagon-like peptide-1 receptor agonists (GLP-1 RA) before the index hospitalization; (4) use of other DPP-4 inhibitors or any GLP-1 RA after the index hospitalization; (5) expiration during index admission; (6) development of composite primary cardiovascular outcome within 1 month of index hospitalization or (7) follow-up for less than 1 month after index hospitalization. The exclusion criteria are shown in Fig. 1.

### Exposure of linagliptin

In the study period, the exposure to linagliptin was based on a computer-based prescription claims after the index hospitalization. Patients with T2DM after ACS or AIS were classified into the linagliptin group or the control group in which patients did not receive incretin-based therapies (any DPP-4 inhibitors or GLP-1 RA) after ACS or AIS.

### Outcomes and covariate measurements

Baseline comorbidities were identified using ICD-9-CM diagnosis codes and medications during the index hospitalization (Tables 1, 2). The primary composite outcome included cardiovascular death, non-fatal myocardial infarction and non-fatal ischemic stroke. The definition of cardiovascular death meet the criteria of Standardized Definitions for End Point Events in Cardiovascular Trials draft by the Food and Drug Administration. Death and causes of death were according to the registry data of NHIRD [32]. Secondary outcomes of interest were death due to any cause, hospitalization for heart failure, percutaneous coronary intervention and coronary artery bypass grafting. Safety outcomes were risks of hypoglycemia, diabetic ketoacidosis, hyperosmolar hyperglycemic state, acute pancreatitis, de novo dialysis, acute hepatitis or newly diagnosed malignancy.

**Table 1:** Characteristics of the study patients before and after propensity score matching

Characteristics	Before matching			After matching	
	Linagliptin (*n* = 401)	Control (*n* = 17,227)	*P*	Control (*n* = 802)	*P*
Age, years	69.6 ± 10.9	70.0 ± 11.1	.525	69.1 ± 11.3	.415
Age ≥ 75 years	143 (35.7)	6216 (36.1)	.862	263 (32.8)	.321
Gender			.081		.744
Male	216 (53.9)	10,029 (58.2)		424 (52.9)	
Female	185 (46.1)	7198 (41.8)		378 (47.1)	
T2DM duration, years	12.9 ± 3.5	11.7 ± 3.9	< .001	12.8 ± 3.4	.922
T2DM duration group (years)			< .001		.825
0–5	20 (5.0)	1508 (8.8)		38 (4.7)	
6–10	54 (13.5)	3064 (17.8)		121 (15.1)	
11–15	212 (52.9)	9425 (54.7)		405 (50.5)	
> 15	115 (28.7)	3230 (18.8)		238 (29.7)	
Comorbidity					
Old myocardial infarction	45 (11.2)	1524 (8.8)	.099	83 (10.3)	.643
Old ischemic stroke	101 (25.2)	4837 (28.1)	.203	229 (28.6)	.217
Heart failure	70 (17.5)	2467 (14.3)	.077	129 (16.1)	.546
Venous thromboembolism	11 (2.7)	241 (1.4)	.025	21 (2.6)	.899
Chronic kidney disease (CKD)			< .001		.469
None	207 (51.6)	11,642 (67.6)		444 (55.4)	
Non-dialysis CKD	161 (40.2)	4479 (26.0)		298 (37.1)	
Dialysis	33 (8.2)	1106 (6.4)		60 (7.5)	
Gout	34 (8.5)	1506 (8.7)	.854	70 (8.7)	.885
Atrial fibrillation	35 (8.7)	1521 (8.8)	.944	54 (6.7)	.213
Peripheral arterial disease	28 (7.0)	1058 (6.1)	.489	59 (7.4)	.813
Hypertension	337 (84.0)	13,851 (80.4)	.069	682 (85.0)	.650
Dyslipidemia	186 (46.4)	6534 (37.9)	.001	350 (43.6)	.367
COPD	30 (7.5)	1534 (8.9)	.322	63 (7.9)	.819
Malignancy	25 (6.2)	1269 (7.4)	.390	58 (7.2)	.520
Cirrhosis	8 (2.0)	356 (2.1)	.921	16 (2.0)	1.000
Autoimmune disease	15 (3.7)	280 (1.6)	.001	34 (4.2)	.680
Previous treatment					
PCI	58 (14.5)	2110 (12.2)	.182	112 (14.0)	.815
CABG	18 (4.5)	419 (2.4)	.009	36 (4.5)	1.000

Values are the mean ± SD or n (%)

*CABG* coronary artery bypass grafting, *COPD* chronic obstructive pulmonary disease, *PCI* percutaneous coronary intervention, *T2DM* type 2 diabetes mellitus

**Table 2:** Medications of the study patients before and after propensity score matching

Characteristics	Before matching			After matching	
	Linagliptin (*n* = 401)	Control (*n* = 17,227)	*P*	Control (*n* = 802)	*P*
Non-DM medication					
Aspirin	341 (85.0)	14,549 (84.5)	.750	682 (85.0)	1.000
Clopidogrel	206 (51.4)	6696 (38.9)	< .001	393 (49.0)	.439
Warfarin	24 (6.0)	1096 (6.4)	.760	47 (5.9)	.931
NOAC	4 (1.0)	197 (1.1)	.785	8 (1.0)	1.000
ACEI/ARB	275 (68.6)	10,829 (62.9)	.019	548 (68.3)	.930
β-blocker	208 (51.9)	7388 (42.9)	< .001	421 (52.5)	.838
CCB	209 (52.1)	8963 (52.0)	.971	431 (53.7)	.595
Digoxin	20 (5.0)	957 (5.6)	.623	45 (5.6)	.652
Statin	218 (54.4)	8048 (46.7)	.002	443 (55.2)	.774
NSAID	99 (24.7)	4321 (25.1)	.857	208 (25.9)	.640
Cox-2 inhibitor	28 (7.0)	1493 (8.7)	.235	54 (6.7)	.871
Diuretic	116 (28.9)	3528 (20.5)	< .001	217 (27.1)	.494
Spironolactone	35 (8.7)	1355 (7.9)	.526	65 (8.1)	.712
Fibrate	32 (8.0)	1242 (7.2)	.556	72 (9.0)	.562
DM medication					
Biguanide	156 (38.9)	8177 (47.5)	.001	331 (41.3)	.430
Sulfonylurea	204 (50.9)	6864 (39.8)	< .001	445 (55.5)	.130
Thiazolidinedione	27 (6.7)	869 (5.0)	.128	47 (5.9)	.553
Alpha-glucosidase inhibitor	81 (20.2)	2037 (11.8)	< .001	165 (20.6)	.879
Non-SU insulin secretagogue (Glinide)	93 (23.2)	2397 (13.9)	< .001	164 (20.4)	.274
Insulin	244 (60.8)	7807 (45.3)	< .001	484 (60.3)	.868

Values are the mean ± SD or n (%)

*ACEI/ARB* angiotensin-converting-enzyme inhibitor/angiotensin receptor blocker, *CCB* calcium channel blocker, *DM* diabetes mellitus, *NOAC* novel oral anticoagulant, *Non-SU* non-sulfonylurea, *NSAID* non-steroidal anti-inflammatory drug

### Statistical analysis

We matched the linagliptin cohort with the control group by a 1:2 ratio based on patient’s characteristics, baseline comorbidities, medication prescribed 90 days since indexed hospitalization (listed in Tables 1, 2), and index year and month by propensity score matching (PSM) to minimize potential selection bias for this cohort study. Clinical characteristics between these two study groups were compared using Chi square test for categorical variables and independent sample t test for continuous variables. Differences between these two study groups in time of the first occurrence of a predefined primary or secondary outcome after index hospitalization were determined by Cox proportional hazard models in which the study group (linagliptin group versus control group) was the only explanatory variable. Time-to-event outcomes were analyzed using predefined periods, including 6 months and until the final follow-up for each study group using the Kaplan–Meier method and log-rank test. A *P* value of less than .05 was considered statistically significant. All data analyses were performed using the SAS version 9.4 (SAS Institute, Cary, NC).

## Results

### Study patients

A total of 1,759,222 T2DM patients were initially enrolled between June 1, 2012 and December 31, 2013, among whom 30,115 T2DM patients were admitted for ACS or AIS. After applying the exclusion criteria, a total of 17,628 T2DM patients aged ≥ 40 years who were hospitalized for ACS or AIS were eligible for our study cohort. After PSM was used to reduce potential confounding and selection bias, the data of 1203 patients were finally included for analyses (Fig. 1).

### Baseline characteristics

Among the 1203 included patients, 401 (33.3%) were in the linagliptin group and 802 matched patients (66.7%) were in the control group. The mean age for the overall cohort was 69.3 years (standard deviation [SD] = 11.2 years). The mean follow-up period was 4.7 months (SD = 2.7 months) and the maximum follow-up time was 15 months. After PSM, the two study groups were well matched in terms of baseline characteristics, comorbidities and non-study medications (Tables 1 and 2). The most common co-morbidity was hypertension (84% vs. 85%), followed by chronic kidney disease (48.4% vs. 44.6%) and dyslipidemia (46.4% vs. 43.6%) in the linagliptin and the control groups, respectively. In addition, patients with old myocardial infarction, heart failure and old ischemic stroke in the linagliptin group were 11.2, 17.5 and 25.2%, respectively; in the control group, those with old myocardial infarction, heart failure and old ischemic stroke were 10.3, 16.1 and 28.6%, respectively (Table 1).

Based on the summary of matched nonstudy medications of anti-diabetic agents (Table 2), both groups had high prevalence of insulin use (linagliptin group: 60.8% and control group: 60.3%), sulfonylurea use (linagliptin group: 50.9% and control group: 55.5%), and alpha-glucosidase inhibitors (linagliptin group: 20.2% and control group: 20.6%).

### Primary outcomes

**Fig. 2:**
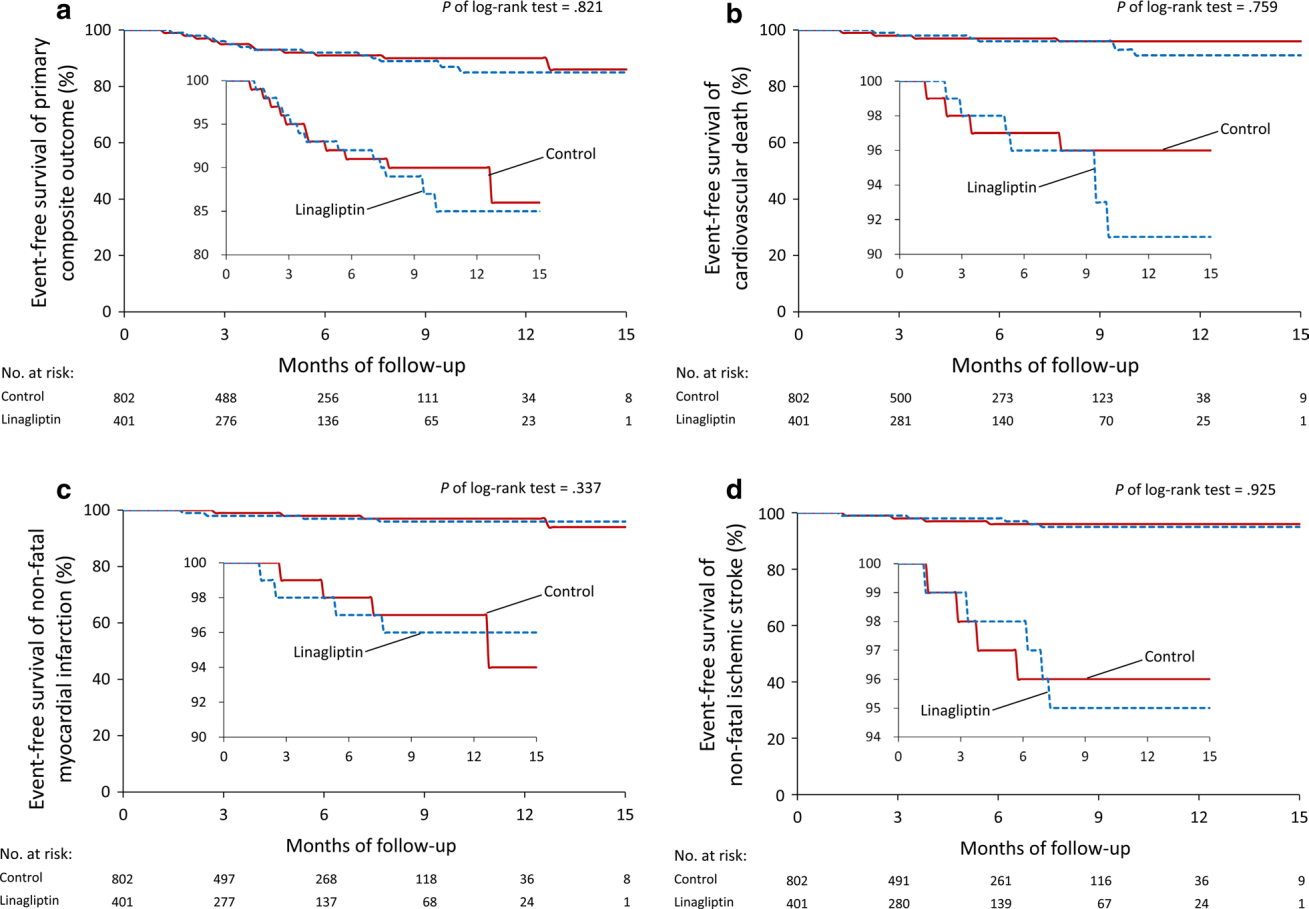
Event-free survival curves in each study group for **a** primary composite outcome, **b** cardiovascular death, **c** non-fatal myocardial infarction, **d** non-fatal ischemic stroke. Primary composite outcome included cardiovascular death, non-fatal myocardial infarction and non-fatal ischemic stroke. No significant differences in the primary composite outcome were observed between these two study groups after a 15-month follow-up

**Fig. 3:**
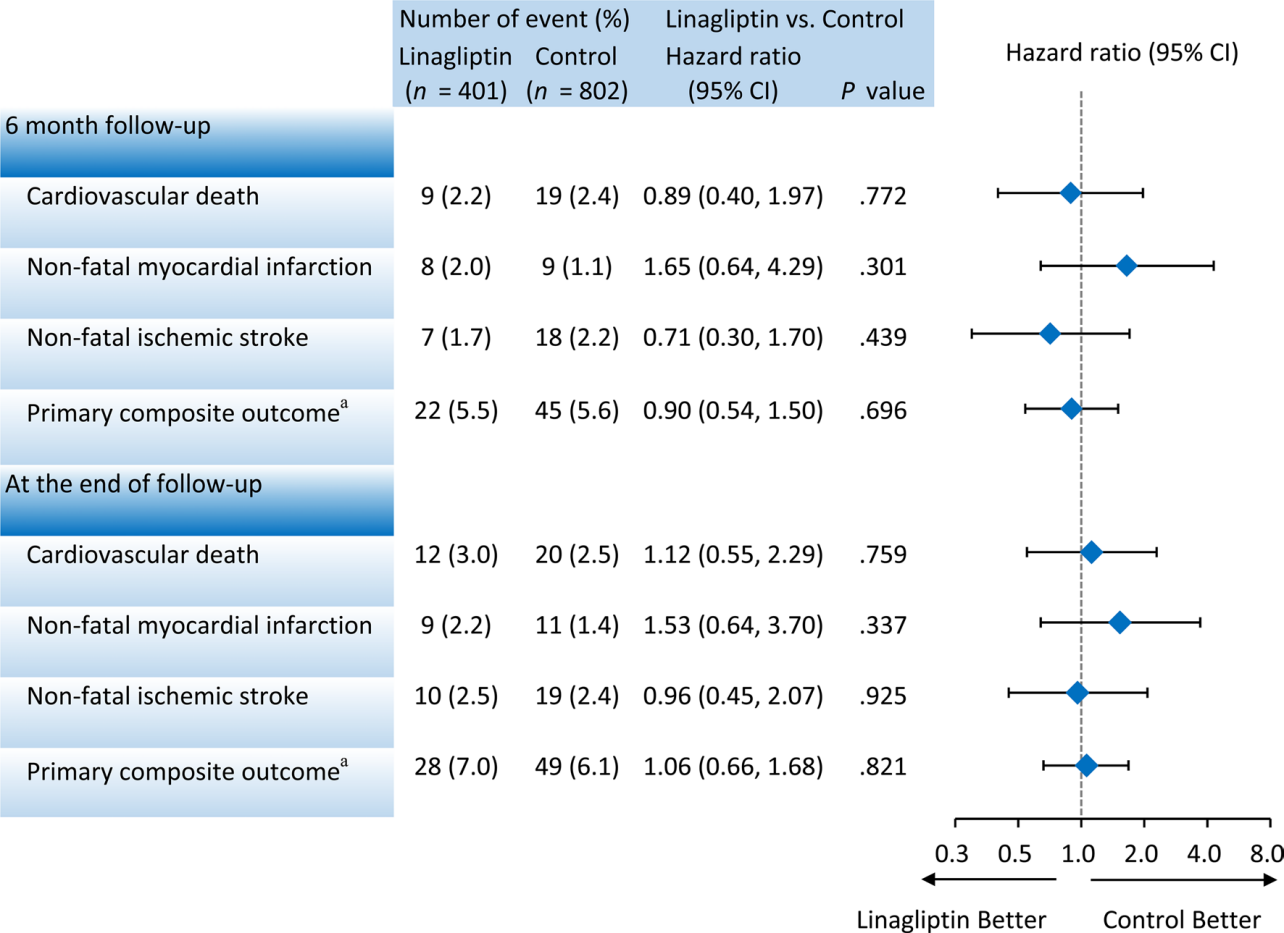
Event numbers and hazard ratio of primary outcomes in all study cohorts

**Fig. 4:**
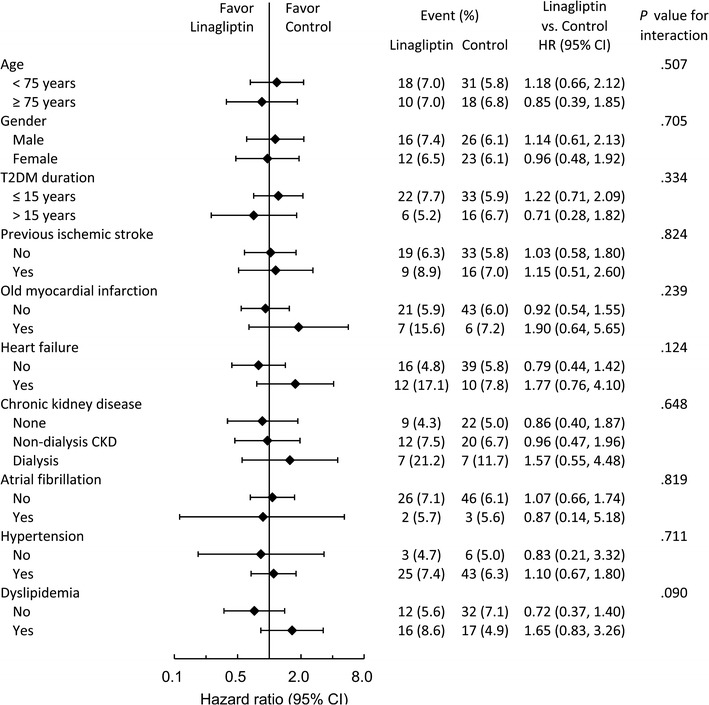
Subgroup analyses for primary composite outcome at the final follow-up

The event-free survival curves of primary composite outcome and each component in these two study groups were plotted (Fig. 2a–d). Events of primary composite outcome occurred in 28 patients (7%) in the linagliptin group and in 49 patients (6.1%) in the control group (HR 1.06; 95% CI .66–1.68) at the final follow-up (Fig. 3). With regard to the individual composite outcome, there was no significant differences in the risks of cardiovascular death (HR 1.12; 95% CI .55–2.29), non-fatal myocardial infarction (HR 1.53; 95% CI .64–3.70) and non-fatal ischemic stroke (HR .96; 95% CI .45–2.07) (Fig. 3). In the subgroup analysis, there were no significant interactions in the prespecified primary cardiovascular outcomes (Fig. 4).

Tables 3 and 4 show primary outcomes in T2DM patients with ACS or AIS, respectively and reveal linagliptin had a neutral effect on cardiovascular death, non-fatal myocardial infarction and non-fatal stroke in either ACS cohort or AIS cohort.

**Table 3:** Primary and secondary outcomes in T2DM with ACS at the end of follow-up

Outcome	Number of event (%)		Linagliptin vs. control	
	Linagliptin (*n* = 154)	Control (*n* = 308)	HR (95% CI)	*P*
Primary outcomes				
6 month follow-up				
Cardiovascular death	5 (3.2)	11 (3.6)	.83 (.29, 2.40)	.735
Non-fatal myocardial infarction	6 (3.9)	8 (2.6)	1.38 (.48, 3.99)	.547
Non-fatal ischemic stroke	1 (.6)	3 (1.0)	.62 (.06, 5.95)	.678
Primary composite outcome^a^	10 (6.5)	22 (7.1)	.84 (.40, 1.78)	.648
At the end of follow-up				
Cardiovascular death	8 (5.2)	13 (4.2)	1.13 (.47, 2.73)	.781
Non-fatal myocardial infarction	7 (4.5)	10 (3.2)	1.29 (.49, 3.38)	.610
Non-fatal ischemic stroke	3 (1.9)	3 (1.0)	1.89 (.38, 9.34)	.438
Primary composite outcome^a^	15 (9.7)	26 (8.4)	1.08 (.57, 2.03)	.821
Secondary outcomes				
All-cause mortality	13 (8.4)	23 (7.5)	1.07 (.54, 2.12)	.840
Other cardiovascular outcomes				
Hospitalization for heart failure	16 (10.4)	22 (7.1)	1.41 (.74, 2.69)	.292
Percutaneous coronary intervention	23 (14.9)	30 (9.7)	1.48 (.86, 2.54)	.160
Coronary artery bypass grafting	4 (2.6)	6 (1.9)	1.25 (.35, 4.44)	.727
Safety outcomes				
Hypoglycemia	6 (3.9)	8 (2.6)	1.43 (.50, 4.12)	.509
DKA or HHS	1 (.6)	2 (.6)	.96 (.09, 10.54)	.970
Acute pancreatitis	0 (.0)	0 (.0)	NA	NA
De novo dialysis	10 (6.5)	16 (5.2)	1.31 (.59, 2.89)	.512
Acute hepatitis	2 (1.3)	0 (.0)	NA	NA
Newly diagnosed malignancy	1 (.6)	5 (1.6)	.38 (.04, 3.25)	.377

*ACS* acute coronary syndrome, *CI* confidence interval, *CV* cardiovascular, *DKA* diabetic ketoacidosis, *HHS* hyperosmolar hyperglycemic state, *HR* hazard ratio, *NA* not applicable, *T2DM* type 2 diabetes mellitus

^a^Anyone of cardiovascular death, non-fatal myocardial infarctionand non-fatal ischemic stroke

**Table 4:** Primary and secondary outcomes in T2DM with AIS at the end of follow-up

Outcome	Number of event (%)		Linagliptin vs. control	
	Linagliptin (*n* = 239)	Control (*n* = 478)	HR (95% CI)	*P*
Primary outcomes				
6 month follow-up				
Cardiovascular death	4 (1.7)	9 (1.9)	.85 (.26, 2.75)	.782
Non-fatal myocardial infarction	0 (.0)	0 (.0)	NA	NA
Non-fatal ischemic stroke	6 (2.5)	24 (5.0)	.47 (.19, 1.14)	.095
Primary composite outcome^a^	10 (4.2)	33 (6.9)	.57 (.28, 1.15)	.114
At the end of follow-up				
Cardiovascular death	4 (1.7)	10 (2.1)	.77 (.24, 2.46)	.658
Non-fatal myocardial infarction	0 (.0)	0 (.0)	NA	NA
Non-fatal ischemic stroke	7 (2.9)	27 (5.6)	.49 (.21, 1.12)	.089
Primary composite outcome^a^	11 (4.6)	36 (7.5)	.57 (.29, 1.12)	.104
Secondary outcomes				
All-cause mortality	9 (3.8)	21 (4.4)	.88 (.40, 1.92)	.742
Other cardiovascular outcomes				
Hospitalization for heart failure	4 (1.7)	7 (1.5)	1.14 (.33, 3.88)	.839
Percutaneous coronary intervention	3 (1.3)	4 (.8)	1.45 (.32, 6.47)	.628
Coronary artery bypass grafting	0 (.0)	0 (.0)	NA	NA
Safety outcomes				
Hypoglycemia	10 (4.2)	18 (3.8)	1.10 (.51, 2.38)	.811
DKA or HHS	2 (.8)	3 (.6)	1.33 (.22, 7.97)	.754
Acute pancreatitis	0 (.0)	0 (.0)	NA	NA
De novo dialysis	8 (3.3)	17 (3.6)	1.20 (.50, 2.86)	.679
Acute hepatitis	0 (.0)	1 (.2)	NA	NA
Newly diagnosed malignancy	7 (2.9)	14 (2.9)	1.32 (.52, 3.39)	.561

*AIS* acute ischemic stroke, *CI* confidence interval, *CV* cardiovascular, *DKA* diabetic ketoacidosis, *HHS* hyperosmolar hyperglycemic state, *HR* hazard ratio, *NA* not applicable, *T2DM* type 2 diabetes mellitus

^a^Anyone of cardiovascular death, non-fatal myocardial infarctionand non-fatal ischemic stroke

### Secondary outcomes

**Fig. 5:**
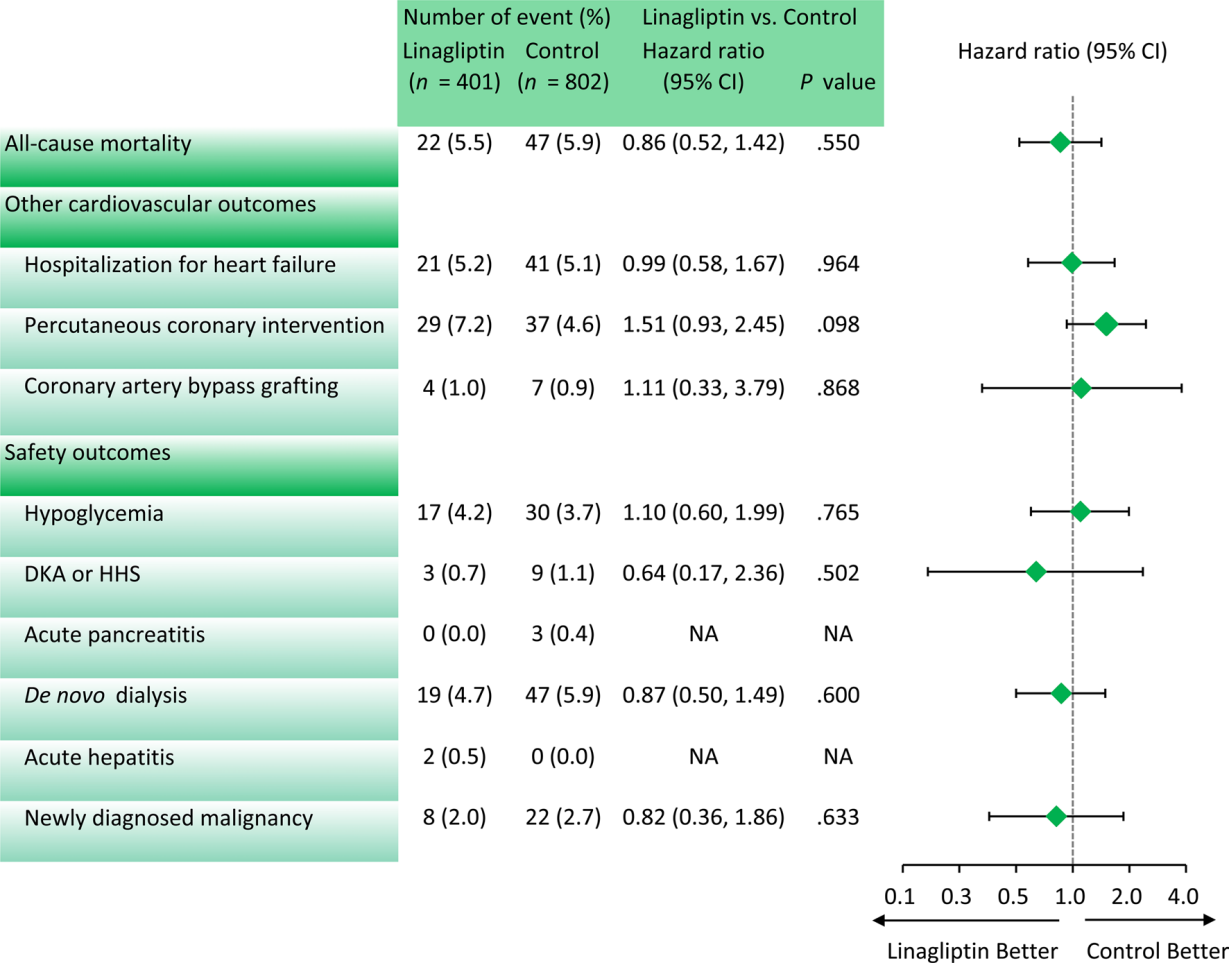
Event numbers and hazard ratio of secondary outcomes in all study cohorts

For secondary outcomes, no significant differences occurred between the linagliptin group and the control group in the respective incidence of all-cause mortality (5.5 and 5.9%; *P* = .550), hospitalization for heart failure (5.2 and 5.1%; *P* = .964), percutaneous coronary intervention (7.2 and 4.6%; *P* = .098) and coronary artery bypass grafting (1.0 and .9%; *P* = .868) (Fig. 5). Tables 3 and 4 show secondary outcomes in T2DM patients with ACS or AIS, respectively and reveal no significant differences in either ACS cohort or AIS cohort.

### Safety outcomes

The linagliptin and control groups did not differ significantly with respect to the incidences of hypoglycemia (4.2 and 3.7%; *P* = .765), diabetic ketoacidosis/hyperosmolar hyperglycemic state (.7 and 1.1%; *P* = .502), acute pancreatitis (0 and .4%; *P* = not applicable), de novo dialysis (4.7 and 5.9%; *P* = .600), acute hepatitis (.5 and 0%; *P* = not applicable), or newly diagnosed malignancy (2.0 and 2.7%; *P* = .633) (Fig. 5).

## Discussion

The strength of our research is that it is the first real world and nationwide population-based study to evaluate the cardiovascular outcomes of linagliptin treatment in T2DM patients after ACS or AIS. The results of our study suggested that short-term use of linagliptin had a neutral effect on composite cardiovascular outcomes in patients with T2DM aged ≥ 40 years after ACS or AIS who were at extremely high risks of further cardiovascular events without increasing the risks of all-cause mortality, hospitalization for heart failure, receiving percutaneous coronary intervention and coronary artery bypass grafting. For safety issues of linagliptin, two well-designed, randomized double-blinded clinical trial, CARMELINA and CAROLINA, respectively, are in progress and results are pending. Nevertheless, both trials exclude patients who suffered from a recent ACS or AIS. Therefore, another valuable strength of our research is that we filled the gap of evidence in this special population with these very high-risk patients who presumably increase the event rate of major cardiovascular diseases to complete the study in a relatively brief period.

### Cardiovascular end points

Several experimental and clinical researches revealed that T2DM patients who were treated with DPP-4 inhibitors had lower risks for cardiovascular diseases as compared to those treated without DPP-4 inhibitors, except for metformin users [33], and DPP-4 inhibitors did not increase the risk of heart failure compared with sulfonylurea [34]. With regard to linagliptin, several studies with limitations of pooled analysis showed that linagliptin in patients with T2DM is not associated with an increase in cardiovascular events [35–38]. The results of our study for primary composite outcome (cardiovascular death, non-fatal myocardial infarction and non-fatal ischemic stroke) are compatible with those of previous randomized controlled trials (i.e., SAVOR [12], EXAMINE [13], and TECOS [14] trials), which indicated that short-term use of DPP-4 inhibitors have a neutral effect on cardiovascular death, myocardial infarction and stroke. Prespecified subgroup analyses in our study suggested that no significant interactions were observed, even in chronic kidney disease (CKD) with or without dialysis. Compared to the results of subgroup analyses in the EXAMINE study which excluded end-stage renal disease (ESRD) patients, the CKD subgroup analyses revealed that alogliptin had an increased primary cardiovascular risk trend in the moderate or severe renal impairment group when compared to the normal or mild renal insufficiency group (*P* value for interaction with treatment = .046) [13].

For issues of hospitalization for heart failure, the SAVOR-TIMI 53 study indicated a 27% increase in hospitalization for heart failure among T2DM patients receiving saxagliptin as compared with those receiving placebo (HR 1.27; 95% CI 1.07–1.51; *P* = .007) [12]. The main predictors of hospitalization for heart failure were previous history of heart failure, elevated brain natriuretic peptide (BNP) and CKD [39]. Compared to patients in the SAVOR study, our patients had more previous history of heart failure (17.5% in the linagliptin group and 16.1% in the control group) than did those with previous history of heart failure in the SAVOR study (12.8% in the saxagliptin group and 12.8% in the placebo group). Besides, unlike the SAVOR study which excluded patients with ESRD receiving long-term dialysis, our study enrolled those with ESRD on dialysis (8.2% in the linagliptin group and 7.5% in the control group). As a result, our result showing that linagliptin had a neutral effect on hospitalization for heart failure could be more convincing and supported that it should not be a class effect of all DPP-4 inhibitors in regards to heart failure.

### Protective effects of vascular diseases

In preclinical studies, linagliptin could prevent female mice from western diet-induced vascular abnormalities [40], and even reverse western diet-induced diastolic dysfunction possibly by targeting TRAF3IP2 expression which is associated with downstream inflammatory signaling [41]. DPP-4 inhibitor-medicated increased GLP-1 may have a direct effect at the neuronal level of brain which was suspected to be associated with the neuroprotective effect on the stroke mice [42].

In clinical studies, the initial combination of linagliptin and metformin substantially enhanced glycemic control without weight gain and with infrequent hypoglycemia [43] and also significantly improved microvascular function in the fasting state [44]. Besides, the special issues of linagliptin is about neuroprotective effects based on some evidences showing that patients treated with linagliptin were significantly associated with fewer events of stroke [25, 26]. Patients with diabetes have a twofold excess risks for ischemic stroke compared with those without diabetes [2] and acute stroke could lead to stress hyperglycemia with increased mortality and poor prognosis [45]. Moreover, stroke-mediated damage could increase the permeability of the BBB which may have a influence on DPP-4 inhibitors for neuroprotection [46]. In the present study, we included patients with recent AIS as a part of the study population. Nevertheless, our results did not find a significant anti-stroke effect with linagliptin treatment at the final follow-up. A 2-year, randomized, double-blind, non-inferiority trial including 1,552 patients by Gallwitz et al. indicated that patients treated with linagliptin had significantly fewer non-fatal stroke than those treated with glimepiride (3 vs. 11 patients; HR .27; 95% CI .08–.97; *P* = .03) [25]. The actual reason for this discrepancy is unclear. However, any of the following explanations may apply. First, the patients in our study conducted in Taiwan were mostly an Asian population. By comparison, the study by Gallwitz et al. only enrolled 12% Asian patients. Because intracranial atherosclerosis is relatively common in Asia [47], the clinical influence of linagliptin may be different in Asian subjects compared to Western subjects. Second, the etiology of ischemic stroke is heterogeneous with large vessel disease, small vessel disease, and embolism which may lead to different effects of linagliptin according to the different etiologies. Third, in our study, we enrolled patients with old ischemic stroke (28.1% in the linagliptin group and 29.5% in the control group) after recent AIS; in contrast, the study by Gallwitz et al. excluded patients with stroke or transient ischemic attack within 6 months before enrollment [25]. Because of the obvious differences in disease severity of the populations, the overall rate of non-fatal ischemic stroke in our study was 2.4% at 15 months but the overall non-fatal stroke rate of the study by Gallwitz et al. was only .9% at 2 years.

## Study limitations

The major limitations of our study are as follows. First, personal information of our patients such as smoking, life style, body mass index, family history of cardiovascular disease or laboratory parameters including levels of glycated hemoglobin were not available. Nevertheless, we were able to include a wide range of variables related to outcomes, including comorbidities and non-study medications, to make our two study groups well balanced. Second, we assumed that patients adhered properly to their treatment medications in the claims data. Third, this is an observational trial and causal effect relationship should be carefully interpreted. Furthermore, it remains unclear whether the findings of our study are applicable to other ethnicities because the population in the present study was in Asia and unique. Our study showed higher use of insulin, sulfonylurea, and alpha-glucosidase inhibitors compared with the United States or Europe. The reasons why the high rates of use with alpha-glucosidase inhibitors, sulfonylurea, and insulin could be as follows. Postprandial hyperglycemia is more common in Asians than in Caucasians [48, 49] and one possible reason could be people in Asia take more carbohydrates and whole grains such as rice or noodles for their meals [50, 51]. Alpha-glucosidase inhibitors which mainly lower postprandial hyperglycemia may have greater effectiveness in Asians than Caucasians and can be a first-line drug according to the guideline of T2DM in China [52–54]. In our present study, we enrolled more patients of CKD with or without dialysis (linagliptin group: 48.4% and control group: 44.6%) because linagliptin is no dosage adjustment required in renal insufficiency including ESRD with dialysis which make it more popular in CKD patients. For patients with non-dialysis CKD who estimated glomerular filtration rate (eGFR) > 25 ml/min/1.73 m^2^, alpha-glucosidase inhibitors (such as acarbose), sulfonylurea (such as glipizide) and insulin are alternative drugs for treatment of T2DM instead of metformin; for patient with dialysis, both sulfonylurea (such as glipizide, still can be used with caution) and insulin could be the main drugs with DPP-IV inhibitors for blood glucose control because other anti-hyperglycemic agents are contraindicated or relatively contraindicated in dialysis patients.

Finally, our study has a mean of 4.7 months and a maximum of 15 months of follow-up because linagliptin was available in Taiwan since 2012. Studies with longer duration of follow-up in the future may provide more information in this special population which is considerably at high cardiovascular risks. Despite these disadvantages, our real-world and nationwide population-based data is still valuable to answer uncertain questions before and after the results of CARMELINA and CAROLINA are published. For these patients at extremely high risks of further cardiovascular events, randomized controlled trials are not always feasible due to considerations of ethical issues, time, or cost.

## Conclusions

In summary, the use of linagliptin in patients with T2DM after ACS or AIS could have a neutral effect on composite cardiovascular events without increasing the risks of all-cause mortality, hospitalization for heart failure, receiving percutaneous coronary intervention, and coronary artery bypass grafting. These results could help clinicians to choose linagliptin as an adequate anti-diabetic agent for T2DM patients at extremely high risks of further cardiovascular events.

## Abbreviations

T2DM: Type 2 diabetes mellitus
ACS: acute coronary syndrome
AIS: acute ischemic stroke
DPP-4: dipeptidyl peptidase-4
GLP-1: glucagon-like peptide-1
CKD: chronic kidney disease
ESRD: end-stage renal disease
PCI: percutaneous coronary intervention
CABG: coronary artery bypass grafting
DKA: diabetic ketoacidosis
HHS: hyperosmolar hyperglycemic state
HR: hazard ratio
CI: confidence interval
